# Generative AI chatbots as sources of phimosis-related consumer health information: a cross-sectional comparison of quality, source transparency, and readability

**DOI:** 10.3389/fpubh.2026.1983152

**Published:** 2026-10-05

**Authors:** Qinghua Luo, Leihua Cao, Pan Ding

**Affiliations:** Department of Urology, Nanchang People’s Hospital, Nanchang, China

**Keywords:** artificial intelligence, chatbot, consumer health information, health literacy, phimosis, readability, urology

## Abstract

**Background:**

Publicly accessible generative artificial intelligence chatbot products are increasingly used for sensitive health questions, but the quality, source transparency, and readability of phimosis-related information remain uncertain.

**Methods:**

We conducted an internet-based cross-sectional comparative study on 1 July 2026. 15 of 25 Google Trends candidate terms were submitted unchanged to ChatGPT (displayed as GPT-5.5), Microsoft Copilot, Gemini (displayed as 3.5 Flash), and Perplexity, yielding 60 responses. Two practising urologists holding senior professional titles, each with more than 10 years of clinical experience, independently assessed responses using DISCERN, Ensuring Quality Information for Patients (EQIP), the Global Quality Score (GQS), and Journal of the American Medical Association (JAMA) benchmark criteria. Inter-rater reliability was assessed using ICC (2,1) for DISCERN and EQIP and quadratic-weighted Cohen’s kappa for JAMA and GQS. Because the same 15 queries were evaluated across all four products, quality outcomes were analysed as matched data using Friedman tests with Kendall’s W and Holm-adjusted paired Wilcoxon signed-rank comparisons. Readability was assessed with six established indices.

**Results:**

Inter-rater reliability was good to almost perfect (coefficients 0.866–0.882). After Holm adjustment across the four omnibus tests, differences remained for DISCERN (*p* = 0.007; *W* = 0.301), EQIP (*p* < 0.001; *W* = 0.587), JAMA (*p* < 0.001; *W* = 0.953), and GQS (*p* = 0.049; *W* = 0.174). For DISCERN, Copilot scored higher than ChatGPT and Gemini, and Perplexity scored higher than Gemini. For EQIP and JAMA, Copilot and Perplexity scored higher than ChatGPT and Gemini. No GQS pairwise comparison remained significant after Holm correction. None of the 15 responses from any product met any individual prespecified readability threshold. A sensitivity analysis excluding the broad standalone prompts ‘circumcision’ and ‘balanitis’ preserved the DISCERN, EQIP, and JAMA findings but not the GQS omnibus difference.

**Conclusion:**

In this purposively derived 15-query, single-date snapshot, chatbot products differed in selected measures of information quality and source transparency, while readability remained consistently above the prespecified sixth-grade target. These findings are conditional on the sampled queries and outputs and should not be interpreted as stable platform-wide estimates of clinical accuracy or safety.

## 1 Introduction

Phimosis describes inability to retract the foreskin sufficiently to expose the glans, but its clinical meaning depends on age and context. Non-retractability is often physiological in young boys, whereas scarring, recurrent inflammation, pain, voiding difficulty, or acquired narrowing may indicate pathological phimosis. Management ranges from reassurance and avoidance of forceful retraction to topical corticosteroids, preputioplasty, or circumcision. Paraphimosis is distinct: a retracted foreskin becomes trapped behind the glans and requires urgent assessment. Current guidance therefore stresses age-specific evaluation and the distinction among normal development, pathological narrowing, inflammatory disease, and emergency presentations (1–3).

Genital symptoms can be embarrassing, and patients or caregivers may seek information anonymously before consulting a clinician. Generative AI chatbots offer immediate conversational answers and are becoming part of routine digital health information seeking. Studies in urology have reported fluent and often broadly informative responses, but they have also identified inconsistent guideline interpretation, limited actionability, weak source attribution, inaccurate or nonfunctional references, and reading levels above those recommended for consumer health information (4–9). These deficiencies are relevant to public health because low-quality or inaccessible digital information can widen health-literacy gaps rather than reduce them.

Comparative chatbot studies in sexually transmitted disease and maintenance hemodialysis have combined DISCERN, EQIP, GQS, JAMA benchmarks, and established readability formulas. Citation-enabled systems often achieved higher relative quality or transparency scores, yet none consistently met plain-language targets (10–13). A coherent response can therefore appear useful while remaining difficult to understand, weakly sourced, or clinically incomplete.

Phimosis warrants focused evaluation because public searches span definitions, symptoms, topical therapy, stretching, circumcision, balanitis, adult disease, and paraphimosis. Poorly framed information may encourage forceful foreskin manipulation, inappropriate self-treatment, unnecessary anxiety, or delayed recognition of an urgent condition. Evidence specifically comparing public-facing chatbot products for phimosis-related consumer information remains limited. We therefore asked whether four widely accessible products differ in information quality, source transparency as operationalized by the JAMA benchmark, and overall educational value, and whether their responses meet a prespecified sixth-grade readability benchmark.

## 2 Materials and methods

### 2.1 Study design and reporting framework

This internet-based cross-sectional comparative evaluation was conducted online from China on 1 July 2026. The study language was English, and the Google Trends geographic setting was worldwide. The unit of analysis was the response to an individual query, with query serving as the matched/repeated-measures unit across the four chatbot products. The 15 retained queries were a purposively derived set of Google Trends entries rather than a probabilistic sample of all possible phimosis-related questions; inferential results are therefore conditional on this selected query set. No patient records, identifiable private information, biological samples, or clinical interventions were involved. Reporting was aligned with the Chatbot Assessment Reporting Tool (CHART) where the available study records permitted (14).

No prospective protocol or registration was documented for this exploratory study. Because the sampling frame was the prespecified set of eligible Google Trends entries rather than a recruited participant population, no *a priori* power calculation was performed.

### 2.2 Identification and selection of phimosis-related queries

The Medical Subject Headings database was consulted to establish the standardized term ‘phimosis.’ Google Trends was accessed on 1 July 2026 for the 5-year period ending on that date, with geography set to Worldwide, and the Top related queries were extracted. Google Trends reports normalized relative interest rather than absolute search volume; relevance values were therefore used to identify commonly sought topics, not to estimate prevalence or population demand (15, 16). The archived study record did not retain whether the Google Trends entry was classified by the interface as a search term or topic, nor the category, search-type, or interface-language settings. These unrecoverable parameters are reported as a reproducibility limitation rather than reconstructed retrospectively.

25 candidate entries were retrieved. Two evaluators independently screened them. Entries were excluded if they were duplicate or highly similar to a retained concept (*n* = 7), primarily navigational/source-oriented rather than health-content queries (‘phimosis reddit’ and ‘phimosis nhs’; *n* = 2), or coding-oriented rather than consumer-facing (‘icd 10 phimosis’; *n* = 1). 15 terms remained. Broad but clinically related terms such as ‘circumcision’ and ‘balanitis’ were retained because they appeared among the Top related searches and represent genuine information-seeking concepts associated with foreskin disorders. To preserve real-world search wording, original English strings were retained rather than expanded into researcher-written questions (Table 1). Because the two broad standalone terms could elicit information not specific to phimosis, a prespecified revision-stage sensitivity analysis repeated the matched quality comparisons after excluding them.

**Table 1:** Google Trends data for the 25 phimosis-related search terms identified worldwide during the 5-year window ending 1 July 2026.

TOP	Relevance
1. Phimosis treatment	100
2. Phimosis meaning	73
3. Phimosis cream	67
4. Paraphimosis	62
5. Phimosis paraphimosis	60
6. Circumcision	54
7. What is phimosis (removed)	54
8. Phimosis circumcision	53
9. Phimosis surgery	44
10. Balanitis	35
11. Cream for phimosis (removed)	32
12. Icd 10 phimosis (removed)	29
13. Steroid cream (removed)	25
14. Phimosis steroid cream	25
15. Phimosis definition (removed)	24
16. Phimosis symptoms	22
17. Phimosis adult	20
18. Phimosis in adults (removed)	19
19. Phimosis cause	18
20. Treatment for phimosis (removed)	18
21. Phimosis reddit (removed)	18
22. Phimosis nhs (removed)	17
23. Phimosis causes (removed)	17
24. Paraphimosis vs. phimosis	17
25. Phimosis stretching	16

Boldface indicates the 15 terms retained for chatbot prompting; “(removed)” indicates excluded entries. Relevance is the normalized Google Trends value, not an absolute search count.

### 2.3 Chatbot selection and response collection

Four publicly accessible chatbot products were evaluated: ChatGPT (OpenAI; displayed as GPT-5.5), Microsoft Copilot, Gemini (Google; displayed as 3.5 Flash), and Perplexity. Each of the 15 retained search strings was submitted once to each product in English without simplification, rewriting, follow-up prompting, or clinician-added context, yielding 60 responses with no missing outputs. A single generation per query was used to capture a standardized cross-sectional snapshot within one collection date and to limit temporal changes in product versions during sampling. This design does not characterize stochastic within-product variability; an unusually structured, detailed, or citation-rich response could increase quality scores, whereas an unusually brief output could lower them. The complete 60 verbatim responses are provided in Supplementary Data S1.

The analysis concerns the user-facing chatbot products as accessed from China on the study date rather than assumed hidden backend models. The archived record preserved the user-visible ChatGPT and Gemini designations and the response content used for scoring, including source-attribution text where present. It did not prospectively record account/login status, free versus paid access, browsing or web-search settings, personalization settings, exact conversation-reset behaviour, geographic routing, or the underlying engines used by Copilot and Perplexity. These parameters therefore cannot be reconstructed with certainty and may have influenced interface behaviour, citation display, or output localization.

### 2.4 Quality, transparency, and educational-value assessment

Two practising urologists holding senior professional titles, each with more than 10 years of clinical experience, independently scored all 60 outputs. Explicit chatbot labels were concealed during scoring. Approximately 10% of responses were used for pilot calibration before formal evaluation. Substantive response content, including citation and source-attribution information required for JAMA and potentially EQIP scoring, was retained; complete platform blinding therefore could not be guaranteed because citation style or response structure could have revealed product identity. Pre-consensus ratings were retained for the revision analysis. Inter-rater reliability was quantified before consensus using a two-way random-effects, absolute-agreement, single-measure intraclass correlation coefficient [ICC (1, 2)] for DISCERN and EQIP and quadratic-weighted Cohen’s kappa for JAMA and GQS. 95% confidence intervals were estimated by percentile bootstrap resampling with 10,000 replicates. Disagreements were then resolved by consensus, and adjudicated scores were used for between-product comparisons. Agreement estimates are summarized in Supplementary Table S1, and response-level scoring records are provided in Supplementary Data S2.

Four instruments assessed distinct properties of the responses. DISCERN evaluates the quality and balance of written health information, especially treatment choices (17). Scores were categorized as very poor (16–26), poor (27–38), fair (39–50), good (51–62), or excellent (63–80). A binary adaptation of the 20-item EQIP checklist was used to evaluate content, presentation, and usability of patient information; each item was scored as yes = 1 or no = 0, and the total was converted to a percentage and classified as excellent (76–100), good (51–75), poor (26–50), or extremely serious quality problems (0–25) (18). GQS provides a global 5-point rating of information flow, overall quality, and usefulness, from 1 (very poor) to 5 (excellent) (19). The four JAMA benchmark criteria assess authorship, attribution, disclosure, and currency; each criterion is scored 0 or 1, for a maximum of 4 (20). No not-applicable category was used in the study’s binary EQIP adaptation, and every instrument item was scored for every response to maintain comparability. Because the query set included definitions, symptoms, treatment, surgery, and emergency-related concepts, this uniform application may disadvantage brief or definition-focused responses, particularly for the treatment-oriented DISCERN instrument.

These instruments evaluate complementary aspects of information quality, presentation, source transparency, and educational utility. In this study, ‘source transparency’ refers specifically to the narrow JAMA domains of authorship, attribution, disclosure, and currency; it does not encompass model training-data provenance, explainability, uncertainty communication, or broader AI transparency. The instruments do not directly establish factual accuracy, clinical safety, empathy, bias, or concordance with phimosis guidelines. Those outcomes were outside the prespecified analysis and were not inferred from the quality scores. Item-level JAMA and EQIP summary results are provided in Supplementary Table S2.

### 2.5 Readability assessment

Before readability analysis, responses were converted to plain text. Formatting symbols, bullet and numbered-list markers, URLs, in-text citation markers, and standalone reference lists were removed without changing substantive wording. Readability was calculated in Python 3.10.4 with textstat 0.7.13.

Six indices were applied: Automated Readability Index (ARI), Gunning Fog Index (GFI), Flesch–Kincaid Grade Level (FKGL), Coleman-Liau Index (CL), Simple Measure of Gobbledygook (SMOG), and Flesch Reading Ease Score (FRES) (21–24). ARI, GFI, FKGL, CL, and SMOG estimate a US school-grade level, with higher values indicating greater complexity. FRES ranges from 0 to 100, with higher values indicating easier text. The prespecified benchmark was FRES ≥ 80 and each grade-level index <6, consistent with commonly used plain-language targets and prior chatbot evaluations (10, 11, 25, 26). The combined criterion was intentionally stringent: it required agreement across formulas that emphasize different text features rather than classifying a response as acceptable on the basis of one index. This composite threshold is a readability convention, not a validated measure of actual patient comprehension.

### 2.6 Statistical analysis

Continuous and ordinal outcomes were reported as median (Q1, Q3). Because the same 15 queries were submitted to all four products, query was treated as the blocking/repeated-measures unit. Overall differences in DISCERN, EQIP, GQS, and JAMA scores were tested using the Friedman test. Kendall’s W was reported as the omnibus effect size, with 95% confidence intervals estimated by query-level percentile bootstrap resampling (10,000 replicates). Because no single primary quality endpoint was preregistered, the four quality analyses were treated as exploratory; the four omnibus *p* values were Holm-adjusted across outcomes. When an omnibus test remained significant, paired Wilcoxon signed-rank tests were used for the six product pairs, with Holm adjustment within each outcome. The statistical inference is conditional on the selected 15-query set and is not intended to represent a random sample of all possible phimosis-related questions.

Readability outcomes were summarized as median (Q1, Q3) and interpreted against the prespecified sixth-grade thresholds (FRES ≥ 80 and <6 for ARI, GFI, FKGL, CL, and SMOG). Response-level attainment was also summarized as n/N (%) for each individual threshold and for simultaneous attainment of all six criteria. No values were imputed because all 60 query-product combinations produced analyzable responses. An exploratory sensitivity analysis repeated the Friedman tests after excluding the broad standalone prompts ‘circumcision’ and ‘balanitis’ (13 matched queries). Statistical tests were two-sided, with *p* < 0.05 considered statistically significant after the specified Holm adjustment. All inferential analyses were performed in Python 3.10.4; readability calculations used textstat 0.7.13.

## 3 Results

### 3.1 Query selection and dataset characteristics

Of the 25 Google Trends entries, 10 were excluded: 7 duplicate or highly similar terms, 2 source-oriented/navigation terms, and 1 coding-oriented term. The 15 retained terms covered treatment, meaning, topical creams, paraphimosis, circumcision, surgery, balanitis, symptoms, adult phimosis, causes, differential terminology, and stretching. Submission of these 15 terms to four products produced 60 responses with no missing outputs. The query set was purposively derived from Google Trends and should be interpreted as a fixed sample of observed search interests rather than a probabilistic sample of all patient questions.

### 3.2 Inter-rater reliability

Agreement between the two independent evaluators was good for DISCERN [ICC (2,1) = 0.877, 95% CI 0.774–0.930] and EQIP [ICC (2,1) = 0.873, 95% CI 0.801–0.925], and almost perfect for JAMA (quadratic-weighted kappa = 0.866, 95% CI 0.729–0.967) and GQS (quadratic-weighted kappa = 0.882, 95% CI 0.771–0.971). The agreement estimates are presented in Supplementary Table S1; response-level independent rater totals are available in Supplementary Data S2.

### 3.3 Quality, transparency, and educational value

Matched analyses showed overall differences among products for all four quality outcomes after Holm adjustment across the omnibus tests (Table 2; Figure 1). DISCERN differed across products (Friedman chi-square = 13.531; Holm-adjusted *p* = 0.007; Kendall’s *W* = 0.301, 95% CI 0.195–0.524). Copilot had the highest median DISCERN score, 45.00 (40.50, 51.50), followed by Perplexity, 43.00 (41.00, 44.00), whereas ChatGPT and Gemini each had a median of 39.00. All four medians remained within the fair DISCERN category.

**Table 2:** Matched quality and source-transparency scores across AI chatbot products.

Outcome	ChatGPT	Copilot	Gemini	Perplexity	Kendall’s W (95% CI)	Holm-adjusted *p*
DISCERN	39.00 (36.50,43.50)	45.00 (40.50,51.50)	39.00 (35.00,43.50)	43.00 (41.00,44.00)	0.301 (0.195–0.524)	0.007
EQIP	55.00 (55.00,62.50)	75.00 (67.50,82.50)	55.00 (55.00,60.00)	75.00 (70.00,75.00)	0.587 (0.399–0.840)	<0.001
JAMA	0.00 (0.00,0.00)	1.00 (1.00,1.00)	0.00 (0.00,0.00)	1.00 (1.00,1.00)	0.953 (0.874–1.000)	<0.001
GQS	4.00 (3.00,4.00)	4.00 (4.00,4.00)	4.00 (3.00,4.00)	3.00 (3.00,4.00)	0.174 (0.050–0.488)	0.049

Values are median (Q1, Q3). *p* values are Holm-adjusted across the four Friedman omnibus tests. Kendall’s *W* is the Friedman effect size; 95% CIs were estimated by query-level percentile bootstrap resampling (10,000 replicates). EQIP, Ensuring Quality Information for Patients; GQS, Global Quality Score; JAMA, Journal of the American Medical Association benchmark criteria.

**Figure 1:**
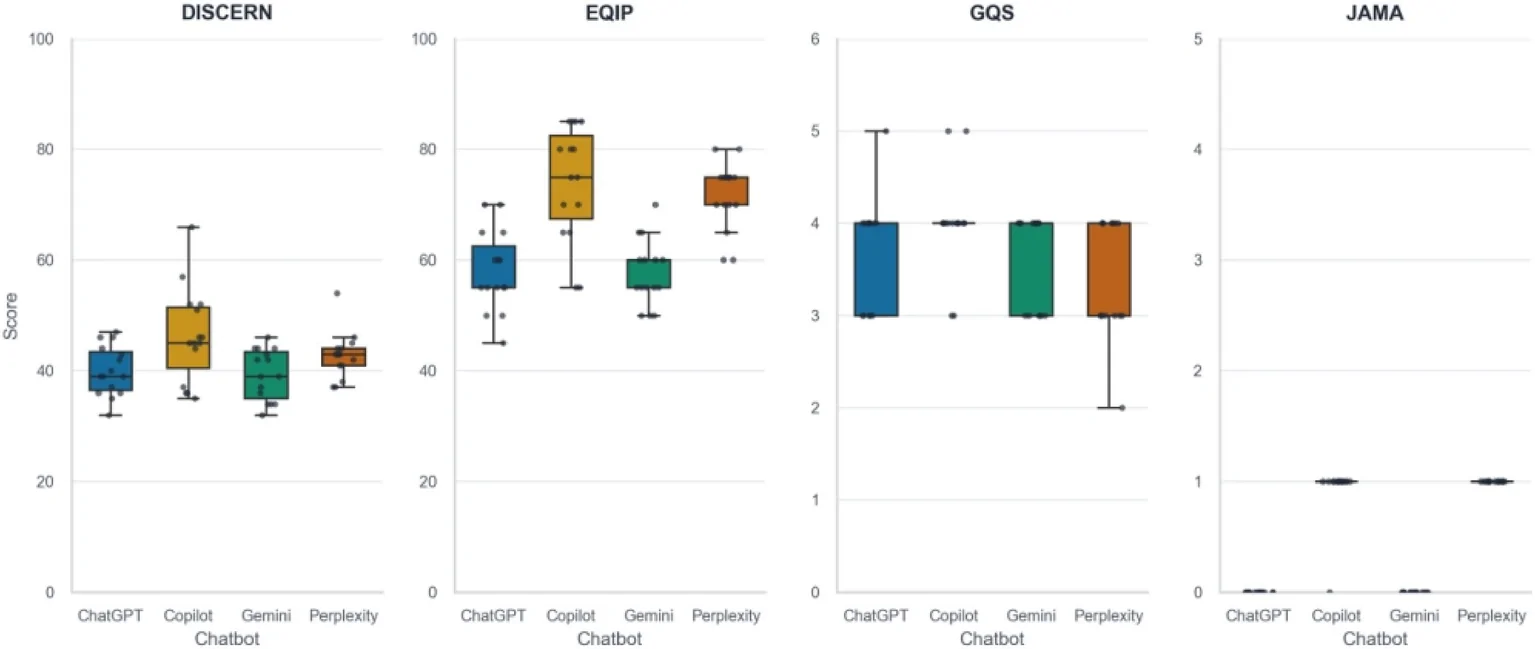
Distribution of DISCERN, EQIP, GQS, and JAMA scores across the four chatbot products. Boxes show interquartile ranges, central lines show medians, and points represent query-level scores.

Differences were larger for EQIP (Friedman chi-square = 26.434; Holm-adjusted *p* < 0.001; *W* = 0.587, 95% CI 0.399–0.840) and JAMA (Friedman chi-square = 42.864; Holm-adjusted *p* < 0.001; *W* = 0.953, 95% CI 0.874–1.000). Copilot and Perplexity each had a median EQIP score of 75.00, compared with 55.00 for ChatGPT and Gemini. Median JAMA scores were 1.00 for Copilot and Perplexity and 0.00 for ChatGPT and Gemini. Item-level inspection showed that the JAMA separation was driven by attribution: 14/15 Copilot responses and 15/15 Perplexity responses met the attribution item, compared with 0/15 for ChatGPT and Gemini; no response from any product met the authorship, disclosure, or currency items. Item-level JAMA and EQIP distributions are provided in Supplementary Table S2. GQS showed a small omnibus difference (Friedman chi-square = 7.839; Holm-adjusted *p* = 0.049; *W* = 0.174, 95% CI 0.050–0.488), but no pairwise GQS comparison remained significant after correction.

### 3.4 Holm-adjusted paired comparisons

Holm-adjusted paired Wilcoxon signed-rank comparisons are presented in Table 3. For DISCERN, Copilot scored higher than ChatGPT (adjusted *p* = 0.028) and Gemini (adjusted *p* = 0.006), while Perplexity scored higher than Gemini (adjusted *p* = 0.028); the remaining DISCERN pairs were not significant. For EQIP, Copilot and Perplexity each scored higher than ChatGPT and Gemini (all adjusted P ≤ 0.006), while Copilot and Perplexity did not differ. JAMA showed the same product separation: Copilot and Perplexity scored higher than ChatGPT and Gemini (all adjusted *p* < 0.001), with no difference between Copilot and Perplexity. No GQS pairwise comparison remained significant after Holm correction.

**Table 3:** Holm-adjusted paired Wilcoxon comparisons of quality scores among AI chatbot products.

Pairwise comparison	DISCERN	EQIP	JAMA	GQS
ChatGPT-Copilot	0.028	0.006	<0.001	0.745
ChatGPT-Gemini	0.493	0.875	1.000	0.745
ChatGPT-Perplexity	0.264	0.006	<0.001	0.235
Copilot-Gemini	0.006	0.006	<0.001	0.176
Copilot-Perplexity	0.297	0.647	0.635	0.176
Gemini-Perplexity	0.028	0.006	<0.001	0.745

Cells show Holm-adjusted *p* values from paired Wilcoxon signed-rank tests. Adjustment was applied to the six pairwise comparisons within each outcome. GQS is shown because its matched omnibus test reached *p* = 0.049, although none of its pairwise comparisons remained significant.

Sensitivity analysis excluding the broad standalone prompts ‘circumcision’ and ‘balanitis’ (13 matched queries) preserved the overall differences for DISCERN (Friedman chi-square = 12.024; Holm-adjusted *p* = 0.015; *W* = 0.308), EQIP (chi-square = 24.504; *p* < 0.001; *W* = 0.628), and JAMA (chi-square = 36.882; *p* < 0.001; *W* = 0.946). The GQS omnibus difference was no longer significant (chi-square = 5.148; Holm-adjusted *p* = 0.161; *W* = 0.132), indicating that the small GQS finding was not robust to exclusion of the two broad terms. Full sensitivity results are provided in Supplementary Table S3.

### 3.5 Readability

Readability profiles differed descriptively across products (Table 4; Figure 2). Gemini produced the highest median ARI (17.00), GFI (17.42), FKGL (15.04), and SMOG (15.73) scores and the lowest median FRES (26.58), whereas Copilot had the highest median CL score (15.76). ChatGPT and Perplexity had lower median grade-level estimates on several indices, but their responses remained above the prespecified target.

**Table 4:** Readability scores across AI chatbot products.

Program	ChatGPT	Copilot	Gemini	Perplexity	6th-grade threshold
ARI	11.44 (10.88,12.54)	13.60 (12.39,14.35)	17.00 (13.79,17.45)	12.18 (10.91,13.46)	<6
GFI	12.81 (11.21,13.97)	14.82 (13.83,16.16)	17.42 (15.31,19.08)	12.83 (11.16,14.12)	<6
FKGL	9.90 (9.18,10.52)	11.45 (10.71,12.44)	15.04 (12.45,15.29)	10.22 (9.22,11.01)	<6
CL	13.32 (12.31,14.12)	15.76 (14.75,17.06)	15.38 (14.16,16.28)	13.97 (11.98,14.26)	<6
SMOG	12.03 (11.24,12.65)	13.09 (12.04,13.95)	15.73 (13.98,16.64)	12.46 (11.54,13.35)	<6
FRES	43.08 (39.74,53.44)	32.63 (26.16,37.46)	26.58 (23.45,37.02)	46.23 (40.80,54.34)	≥80

Values are median (Q1, Q3). Sixth-grade thresholds were FRES ≥ 80 and <6 for ARI, GFI, FKGL, CL, and SMOG. For each chatbot product, 0/15 responses (0%) met each individual threshold and 0/15 met all six simultaneously. ARI, Automated Readability Index; GFI, Gunning Fog Index; FKGL, Flesch–Kincaid Grade Level; CL, Coleman-Liau Index; SMOG, Simple Measure of Gobbledygook; FRES, Flesch Reading Ease Score.

**Figure 2:**
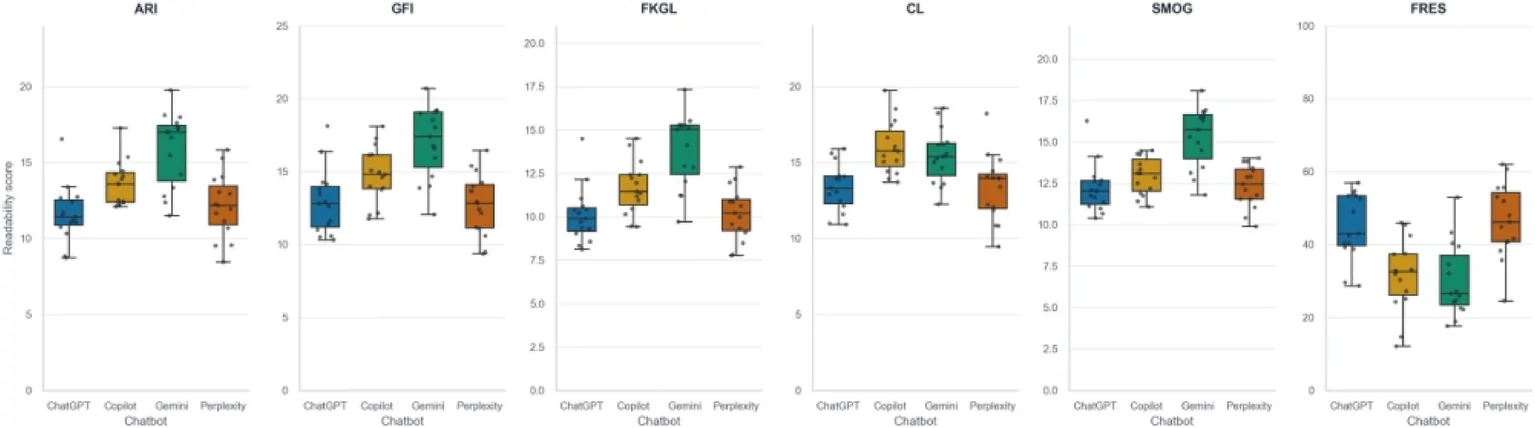
Distribution of six readability indices across the four chatbot products. Higher ARI, GFI, FKGL, CL, and SMOG scores indicate more difficult text; lower FRES values indicate more difficult text.

At the response level, none of the 15 outputs from any product met any individual prespecified readability criterion: ARI < 6, GFI < 6, FKGL <6, CL < 6, SMOG <6, or FRES ≥ 80 were each achieved by 0/15 responses (0%) for ChatGPT, Copilot, Gemini, and Perplexity. Consequently, 0/15 responses (0%) from each product met all six criteria simultaneously. Response-level attainment results are provided in Supplementary Table S4.

### 3.6 Query-level variability

Scores varied by query within every product (Figure 3). Copilot and Perplexity more consistently achieved higher EQIP and JAMA values, whereas Gemini more consistently produced high grade-level values and low FRES values. These within-product differences are important because the 15 queries constitute the matched inferential unit: a product-level median cannot guarantee the quality or accessibility of an answer to an individual phimosis-related search, and the observed differences should not be generalized beyond the sampled query set without further testing.

**Figure 3:**
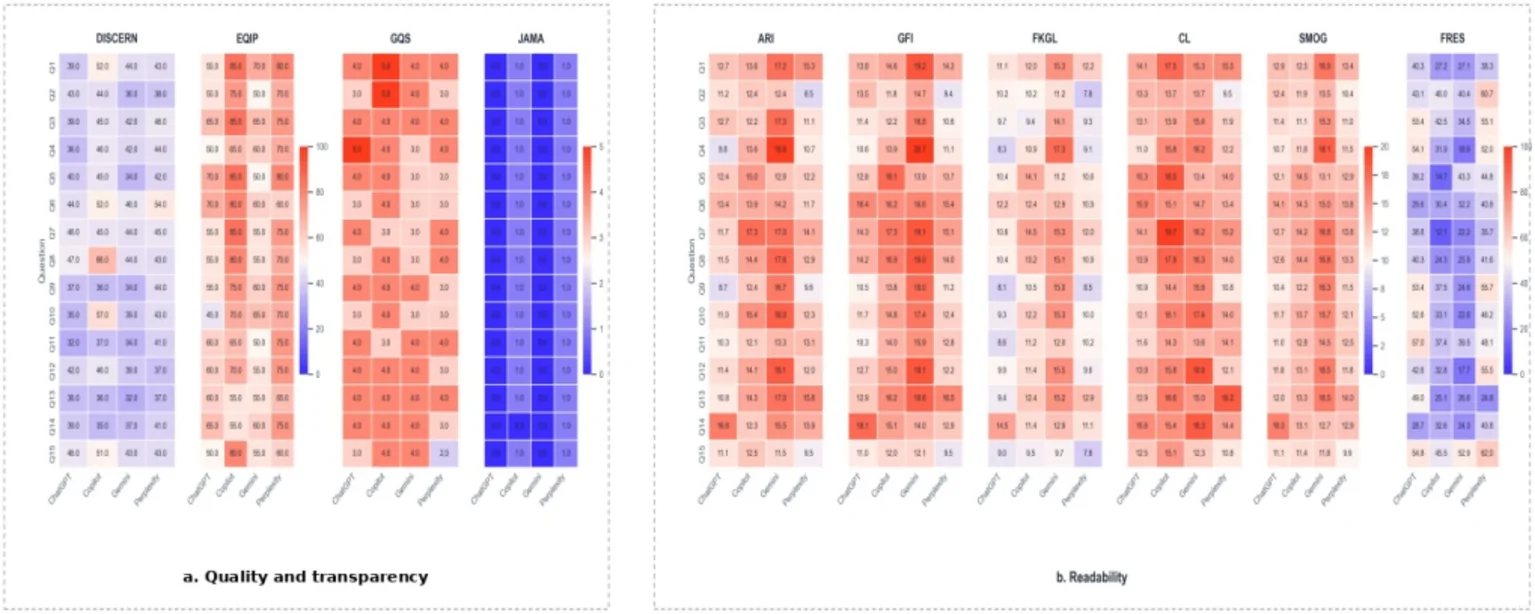
Query-level heatmaps for **(a)** quality and source-transparency outcomes and **(b)** readability outcomes. Q1–Q15 follow the order of the 15 boldface retained terms in Table 1.

## 4 Discussion

### 4.1 Principal findings

Within this matched 15-query snapshot, the four chatbot products differed in selected measures of information quality and source transparency, while readability was consistently more complex than the prespecified sixth-grade target. Copilot had the highest median DISCERN score, and both Copilot and Perplexity had higher EQIP and JAMA scores than ChatGPT and Gemini in the paired analyses. The GQS omnibus result was small and borderline after multiplicity adjustment, no GQS pairwise comparison was significant, and the GQS result disappeared after exclusion of the two broad standalone prompts. Absolute performance remained limited: all median DISCERN scores were fair, median JAMA scores were no higher than 1 of 4, and no individual response met any of the six readability thresholds.

These findings do not establish that one product gives more factually accurate or safer phimosis advice than another. DISCERN, EQIP, GQS, and JAMA assess organization, balance, presentation, usefulness, and selected source-transparency features. Direct factual accuracy, clinical safety, empathy, bias, and guideline concordance were not scored. Statistical inference is also conditional on the purposively selected Google Trends query set rather than a random sample of all phimosis-related information needs.

### 4.2 Comparison with previous chatbot evaluations

Our findings are consistent with comparative studies that have reported stronger quality or transparency scores for citation-oriented systems while also identifying persistent readability problems. Yildiz and Sogutdelen found that Perplexity and Copilot generally outperformed ChatGPT and Gemini for sexually transmitted disease information, although none met recommended reading levels (10). Cao et al. (11) similarly reported higher DISCERN, EQIP, and JAMA scores for Perplexity in maintenance hemodialysis, while readability remained above recommended targets across models.

Urological evaluations also show a recurrent gap between conversational fluency and patient-facing utility. Studies of urological malignancies and common urological conditions have reported useful general explanations alongside limitations in actionability, guideline interpretation, and reference quality (6–9). The present study extends this pattern to a common and sensitive genital-health topic with age-dependent management and a time-sensitive differential diagnosis.

### 4.3 Interpretation of quality and transparency differences

The higher EQIP and JAMA scores for Copilot and Perplexity may reflect product features that make organization or source attribution more visible. Item-level JAMA results support this interpretation: attribution was present in 14/15 Copilot and 15/15 Perplexity responses but in none of the ChatGPT or Gemini responses, while authorship, disclosure, and currency were absent across all four products (Supplementary Table S2). Thus, the JAMA difference was driven almost entirely by attribution behaviour. In this study, JAMA should therefore be interpreted as a narrow measure of source transparency rather than a comprehensive assessment of AI transparency, which could also include model provenance, training-data disclosure, uncertainty communication, or explainability. A citation-enabled interface can improve JAMA scores without proving greater clinical accuracy.

GQS provided a different pattern. The matched omnibus test reached the nominal threshold after Holm adjustment, but the effect was small, no corrected pairwise comparison was significant, and the result was not retained when the broad standalone prompts ‘circumcision’ and ‘balanitis’ were excluded. We therefore do not interpret GQS as evidence of stable product separation. This illustrates the value of query-level blocking and sensitivity analysis when a small, purposively derived prompt set is used.

### 4.4 Public-health and health-literacy implications

Phimosis-related search strings are brief and often omit the context needed for safe counseling. In a child, non-retractability may be physiological and require reassurance; in an adult, newly acquired narrowing can reflect inflammation, scarring, dermatological disease, or other pathology. Topical corticosteroids can improve resolution in boys, but appropriate use depends on diagnosis, age, technique, and follow-up (2). Consumer-facing systems should therefore establish basic context before presenting management options.

Paraphimosis is especially important in public-facing information because a trapped retracted foreskin may require urgent care. Although safety was not formally scored in this study, the inclusion of search terms such as “paraphimosis” means that future evaluations should directly test whether chatbots recognize emergency features and avoid advice that could delay assessment. That question requires a prespecified clinical reference standard rather than extrapolation from general quality scores.

All four products had readability profiles above the sixth-grade target, and none of the 60 individual responses met even one of the six prespecified thresholds. Formula-based readability measures, however, are imperfect proxies for comprehension. Unavoidable domain terms such as phimosis, paraphimosis, balanitis, corticosteroid, and preputioplasty can increase syllable- or word-length-based scores. We did not remove such terms and recalculate the indices because doing so would alter the authentic text presented to users. Even at the most favorable response level, the maximum FRES was 62.01, while the minimum observed ARI, GFI, FKGL, CL, and SMOG values were 8.46, 9.38, 7.77, 9.46, and 9.89, respectively, all outside the prespecified thresholds. Removal of several technical terms might improve these estimates, but whether it would change threshold classification was not tested. Accordingly, the present data cannot quantify how much of the estimated difficulty is attributable specifically to medical terminology. Sentence length, lexical complexity, and response structure may also contribute, but this study did not isolate their independent effects.

Readability formulas are proxies rather than direct tests of comprehension. A numerically simpler answer may omit essential risk information, while a more complex answer may still be understood by a highly literate user. The stringent combined threshold used here should therefore be read as a plain-language screening benchmark rather than a clinical comprehension endpoint. Future studies should combine readability indices with user testing, comprehension measures, actionability assessment, and direct scoring of clinical accuracy and safety (25–27).

### 4.5 Implications for patients, clinicians, and developers

Patients and caregivers should treat chatbot output as supplementary orientation rather than a diagnosis or treatment plan. Clinicians can ask about information obtained from AI systems, correct misconceptions, and provide reviewed plain-language materials when counseling patients about foreskin development, topical treatment, surgery, or warning symptoms.

For developers, phimosis-related systems should distinguish age and clinical context, recognize emergency presentations, and make sources visible, dated, and traceable. Patient-facing responses should define unavoidable medical terms and present conservative and surgical options in balanced language. These product goals should be evaluated directly in future studies rather than assumed from general chatbot capability.

### 4.6 Strengths and limitations

This study applied the same 15 public search terms to four contemporary chatbot products, used matched statistical methods at the query level, and evaluated treatment-information quality, patient-information presentation, global usefulness, source transparency, and six readability dimensions. Independent clinician ratings showed good to almost perfect inter-rater reliability. Reporting matched effect sizes with confidence intervals, multiplicity-adjusted paired comparisons, response-level readability attainment, sensitivity analysis, and query-level heatmaps reduced reliance on a single platform-level ranking.

Several limitations constrain interpretation. First, this was a single-date snapshot on 1 July 2026. Each query was generated once per product, so stochastic within-product variability was not sampled; a particularly structured or citation-rich output could overestimate a product’s typical DISCERN, EQIP, or JAMA performance, whereas an unusually brief output could underestimate it. Second, the 15 Google Trends queries were purposively derived rather than randomly sampled and leaned toward definitions and management. They do not represent the full range of phimosis-related concerns, including psychological distress, body image, sexual function, partner concerns, or cultural and religious contexts of circumcision. Third, DISCERN was designed primarily for treatment-choice information, and applying the full instrument uniformly to heterogeneous query intentions may penalize appropriate short definitional answers; the binary EQIP adaptation used here reduces the response granularity of the original instrument; and JAMA captures only selected source-transparency features. Fourth, Google Trends settings were incompletely archived, and platform account, browsing, personalization, session-isolation, backend-engine, access-route, and geographic-routing details could not be fully reconstructed. Fifth, explicit product labels were concealed during scoring, but citation styles and response structures may have made complete blinding impossible. Sixth, only English outputs were analyzed. Finally, the study did not directly measure factual accuracy, clinical safety, empathy, bias, guideline concordance, or real-user comprehension. These boundary conditions mean that the results describe the sampled outputs rather than stable, universal properties of the chatbot products.

## 5 Conclusion

In this purposively derived, matched 15-query snapshot, public-facing chatbot products differed in selected measures of information quality and source transparency. Copilot and Perplexity performed better on several paired DISCERN, EQIP, and JAMA comparisons, but all median DISCERN scores remained fair and the JAMA separation was driven principally by attribution. The small GQS omnibus finding was not supported by corrected pairwise comparisons or by the sensitivity analysis. None of the 60 individual responses met any of the six prespecified readability thresholds. These products may serve as supplementary orientation tools, but the present findings should not be generalized to all phimosis-related questions or interpreted as evidence of superior clinical accuracy or safety. Repeated-generation studies with broader query sets, direct guideline-based accuracy and safety assessment, and user comprehension testing are needed before stronger claims about patient-facing clinical utility can be made.

## Data Availability

The datasets presented in this study can be found in online repositories. The names of the repository/repositories and accession number(s) can be found in the article/Supplementary material.
