# Supplementary material for: Generative AI chatbots as sources of phimosis-related consumer health information: a cross-sectional comparison of quality, source transparency, and readability
Source: Front Public Health. 2026 Oct 5;14:1983152. doi: 10.3389/fpubh.2026.1983152 (PMC13677900; doi:10.3389/fpubh.2026.1983152)
Supplement: Supplementary file 1 [file Table_3.docx]

**Supplementary Tables S1-S4**

**Supplementary Table S1. Inter-rater agreement for clinician-rated information-quality outcomes**

| **Outcome** | **Agreement statistic** | **Coefficient** | **95% CI** | **Interpretation** |
| --- | --- | --- | --- | --- |
| DISCERN total score | ICC(2,1) | 0.877 | 0.774-0.930 | Good |
| EQIP total score | ICC(2,1) | 0.873 | 0.801-0.925 | Good |
| JAMA total score | Quadratic-weighted Cohen's κ | 0.866 | 0.729-0.967 | Almost perfect |
| GQS total score | Quadratic-weighted Cohen's κ | 0.882 | 0.771-0.971 | Almost perfect |

Note. DISCERN and EQIP were assessed using a two-way random-effects, absolute-agreement, single-measure intraclass correlation coefficient [ICC(2,1)]. JAMA and GQS were assessed using quadratic-weighted Cohen's κ. Ninety-five percent confidence intervals were estimated using percentile bootstrap resampling with 10,000 replicates.

**Supplementary Table S2. Item-level JAMA benchmark and EQIP results across chatbot products**

**Panel A. JAMA benchmark items**

| **JAMA item** | **ChatGPT** | **Copilot** | **Gemini** | **Perplexity** |
| --- | --- | --- | --- | --- |
| J1 Authorship | 0/15 (0.0%) | 0/15 (0.0%) | 0/15 (0.0%) | 0/15 (0.0%) |
| J2 Attribution | 0/15 (0.0%) | 14/15 (93.3%) | 0/15 (0.0%) | 15/15 (100.0%) |
| J3 Disclosure | 0/15 (0.0%) | 0/15 (0.0%) | 0/15 (0.0%) | 0/15 (0.0%) |
| J4 Currency | 0/15 (0.0%) | 0/15 (0.0%) | 0/15 (0.0%) | 0/15 (0.0%) |

Values are n/15 (%), indicating the number and proportion of responses satisfying each JAMA benchmark item.

**Panel B. EQIP item-level attainment**

| **EQIP item** | **ChatGPT** | **Copilot** | **Gemini** | **Perplexity** |
| --- | --- | --- | --- | --- |
| E1 | 0/15 (0.0%) | 14/15 (93.3%) | 0/15 (0.0%) | 15/15 (100.0%) |
| E2 | 0/15 (0.0%) | 14/15 (93.3%) | 0/15 (0.0%) | 15/15 (100.0%) |
| E3 | 15/15 (100.0%) | 15/15 (100.0%) | 15/15 (100.0%) | 15/15 (100.0%) |
| E4 | 15/15 (100.0%) | 15/15 (100.0%) | 15/15 (100.0%) | 14/15 (93.3%) |
| E5 | 1/15 (6.7%) | 14/15 (93.3%) | 0/15 (0.0%) | 15/15 (100.0%) |
| E6 | 0/15 (0.0%) | 0/15 (0.0%) | 0/15 (0.0%) | 0/15 (0.0%) |
| E7 | 14/15 (93.3%) | 11/15 (73.3%) | 12/15 (80.0%) | 13/15 (86.7%) |
| E8 | 1/15 (6.7%) | 3/15 (20.0%) | 1/15 (6.7%) | 1/15 (6.7%) |
| E9 | 15/15 (100.0%) | 15/15 (100.0%) | 15/15 (100.0%) | 15/15 (100.0%) |
| E10 | 15/15 (100.0%) | 15/15 (100.0%) | 15/15 (100.0%) | 15/15 (100.0%) |
| E11 | 15/15 (100.0%) | 15/15 (100.0%) | 15/15 (100.0%) | 15/15 (100.0%) |
| E12 | 15/15 (100.0%) | 15/15 (100.0%) | 15/15 (100.0%) | 15/15 (100.0%) |
| E13 | 14/15 (93.3%) | 12/15 (80.0%) | 12/15 (80.0%) | 13/15 (86.7%) |
| E14 | 9/15 (60.0%) | 15/15 (100.0%) | 15/15 (100.0%) | 15/15 (100.0%) |
| E15 | 10/15 (66.7%) | 9/15 (60.0%) | 8/15 (53.3%) | 5/15 (33.3%) |
| E16 | 12/15 (80.0%) | 11/15 (73.3%) | 13/15 (86.7%) | 10/15 (66.7%) |
| E17 | 15/15 (100.0%) | 15/15 (100.0%) | 15/15 (100.0%) | 15/15 (100.0%) |
| E18 | 0/15 (0.0%) | 0/15 (0.0%) | 0/15 (0.0%) | 0/15 (0.0%) |
| E19 | 4/15 (26.7%) | 8/15 (53.3%) | 6/15 (40.0%) | 6/15 (40.0%) |
| E20 | 4/15 (26.7%) | 6/15 (40.0%) | 1/15 (6.7%) | 3/15 (20.0%) |

Values are n/15 (%), indicating the number and proportion of responses satisfying each EQIP item. Item numbers correspond to the 20-item EQIP scoring instrument used in the study.

**Supplementary Table S3. Sensitivity analysis excluding the broad standalone prompts "circumcision" and "balanitis"**

| **Outcome** | **Matched queries** | **Friedman χ²** | **Kendall's W** | **Raw P** | **Holm-adjusted omnibus P** |
| --- | --- | --- | --- | --- | --- |
| DISCERN | 13 | 12.024 | 0.308 | 0.007 | 0.015 |
| EQIP | 13 | 24.504 | 0.628 | <0.001 | <0.001 |
| JAMA | 13 | 36.882 | 0.946 | <0.001 | <0.001 |
| GQS | 13 | 5.148 | 0.132 | 0.161 | 0.161 |

Note. The same matched-query Friedman framework as the primary analysis was repeated after excluding the two broad standalone prompts. Holm adjustment was applied across the four omnibus tests.

**Supplementary Table S4. Response-level attainment of prespecified sixth-grade readability thresholds**

| **Chatbot product** | **ARI <6** | **GFI <6** | **FKGL <6** | **CL <6** | **SMOG <6** | **FRES ≥80** | **All six criteria** |
| --- | --- | --- | --- | --- | --- | --- | --- |
| ChatGPT | 0/15 (0.0%) | 0/15 (0.0%) | 0/15 (0.0%) | 0/15 (0.0%) | 0/15 (0.0%) | 0/15 (0.0%) | 0/15 (0.0%) |
| Copilot | 0/15 (0.0%) | 0/15 (0.0%) | 0/15 (0.0%) | 0/15 (0.0%) | 0/15 (0.0%) | 0/15 (0.0%) | 0/15 (0.0%) |
| Gemini | 0/15 (0.0%) | 0/15 (0.0%) | 0/15 (0.0%) | 0/15 (0.0%) | 0/15 (0.0%) | 0/15 (0.0%) | 0/15 (0.0%) |
| Perplexity | 0/15 (0.0%) | 0/15 (0.0%) | 0/15 (0.0%) | 0/15 (0.0%) | 0/15 (0.0%) | 0/15 (0.0%) | 0/15 (0.0%) |

Note. Values are n/15 (%). ARI, Automated Readability Index; GFI, Gunning Fog Index; FKGL, Flesch-Kincaid Grade Level; CL, Coleman-Liau Index; SMOG, Simple Measure of Gobbledygook; FRES, Flesch Reading Ease Score.
